# Severe syndromic ID and skewed X-inactivation in a girl with *NAA10* dysfunction and a novel heterozygous de novo *NAA10* p.(His16Pro) variant - a case report

**DOI:** 10.1186/s12881-020-01091-1

**Published:** 2020-07-22

**Authors:** Ingrid Bader, Nina McTiernan, Christine Darbakk, Eugen Boltshauser, Rasmus Ree, Sabine Ebner, Johannes A. Mayr, Thomas Arnesen

**Affiliations:** 1 Einheit für Klinische Genetik, Universitätsklinik für Kinder- und Jugendheilkunde, Paracelsus Medizinische Universität, Müllner Hauptstraße 48, A-5020 Salzburg, Austria; 2 Department of Biomedicine, University of Bergen, Bergen, Norway; 3 Children’s University Hospital, Zürich, Switzerland; 4 Children’s Hospital, Paracelsus Medical University, Salzburg, Austria; 5 Department of Biological Sciences, University of Bergen, Bergen, Norway; 6 Department of Surgery, Haukeland University Hospital, Bergen, Norway

**Keywords:** NAA10, X-linked intellectual disability (XLID), N-alpha-acetyltransferase, Acetylation, NatA, Case report

## Abstract

**Background:**

NAA10 is the catalytic subunit of the major N-terminal acetyltransferase complex NatA which acetylates almost half the human proteome. Over the past decade, many NAA10 missense variants have been reported as causative of genetic disease in humans. Individuals harboring NAA10 variants often display variable degrees of intellectual disability (ID), developmental delay, and cardiac anomalies. Initially, carrier females appeared to be oligo- or asymptomatic with X-inactivation pattern skewed towards the wild type allele. However, recently it has been shown that NAA10 variants can cause syndromic or non-syndromic intellectual disability in females as well. The impact of specific NAA10 variants and the X-inactivation pattern on the individual phenotype in females remains to be elucidated.

**Case presentation:**

Here we present a novel de novo *NAA10* (NM_003491.3) c.[47A > C];[=] (p.[His16Pro];[=]) variant identified in a young female. The 10-year-old girl has severely delayed motor and language development, disturbed behavior with hyperactivity and restlessness, moderate dilatation of the ventricular system and extracerebral CSF spaces. Her blood leukocyte X-inactivation pattern was skewed (95/5) towards the maternally inherited X-chromosome. Our functional study indicates that NAA10 p.(H16P) impairs NatA complex formation and NatA catalytic activity, while monomeric NAA10 catalytic activity appears to be intact. Furthermore, cycloheximide experiments show that the NAA10 H16P variant does not affect the cellular stability of NAA10.

**Discussion and conclusions:**

We demonstrate that NAA10 p.(His16Pro) causes a severe form of syndromic ID in a girl most likely through impaired NatA-mediated Nt-acetylation of cellular proteins. X-inactivation analyses showed a skewed X-inactivation pattern in DNA from blood of the patient with the maternally inherited allele being preferentially methylated/inactivated.

## Background

A majority of the human proteome is N-terminal acetylated by a group of enzymes named N-terminal (Nt) acetyltransferases (NATs). Eight NATs have been identified so far (NatA-NatH), with NatA-NatF and NatH being expressed in humans [1]. NatA is the major NAT responsible for co-translationally Nt-acetylating nearly half of the human proteome [2]. NatA is composed of the evolutionarily conserved catalytic subunit NAA10 and the auxiliary subunits NAA15, NAA50 and HYPK [3–5]. NAA15 is responsible for anchoring the NatA complex to the ribosome as well as modulating the substrate specificity of NAA10 [6–8]. NatA targets small amino acids like Ser and Thr at the N-termini after the initiator methionine has been removed [2]. Furthermore, there is also a cellular pool of NAA10 that is not bound to NAA15 which is suggested to exert distinct roles in the cell [9]. Firstly, several studies have suggested that NAA10 has lysine acetyltransferase (KAT) activity, catalyzing the acetylation of internal lysines of a number of substrate targets including Hsp70 and beta-catenin [10–12]. Secondly, NAA10 has been reported to regulate substrates in a non-catalytic manner. For instance, NAA10 has a role in genomic imprinting through direct binding of non-methylated DNA motifs and recruitment of DNMT1 [13, 14]. Finally, monomeric NAA10 also displays NAT activity distinct of its NatA activity in preferring acidic N-termini in vitro [9]. However, no in vivo NAT substrates of monomeric NAA10 have been identified so far. *NAA10* is an essential gene and loss of NAA10 is associated with developmental defects and lethality in model organisms [15–18].

The human *NAA10* gene is located in Xq28, and several hereditary or de novo NAA10 variants have been reported to be pathogenic in humans [19]. Originally, a missense variant NAA10 p.(Ser37Pro) was identified in eight males from two families with Ogden syndrome (OMIM#300855) [20]. The affected boys died between ages 5 and 16 months mainly because of cardiac abnormalities [20]. Their unaffected carrier-mothers showed a skewed X-inactivation pattern [21]. The NAA10 p.(Ser37Pro) variant was found to impair both NatA complex formation and NatA catalytic activity [21]. Since the discovery of Ogden syndrome, various pathogenic NAA10 variants have been reported in both males and females. A NAA10 p.(Tyr43Ser) variant was identified in two brothers with syndromic intellectual disability (ID) and long QT [22]. Their mildly affected mother was a heterozygous carrier, and X-inactivation studies showed a normal non-skewed (random) inactivation pattern in her blood. Two brothers and an unrelated male with developmental delay (DD), ID and cardiac abnormalities were found to harbor a NAA10 p.(Ile72Thr) variant [23]. Five NAA10 variants, p.(Val107Phe), p.(Phe128Leu), p.(Phe128Ile), p.(Val111Gly), and p.(Arg116Trp) have been reported in unrelated girls with random X-inactivation patterns in lymphocytes and varying degrees of ID [24–26]. Eighteen females with ID and DD have been found to harbor a NAA10 p.(Arg83Cys) variant, which makes it the most commonly reported NAA10 variant to date [25]. Furthermore, a NAA10 p.(Arg83His) variant has been reported in two unrelated boys with ID, DD and hypertrophic cardiomyopathy [27]. A recent comprehensive cohort presented 23 individuals harboring ten different NAA10 variants, whereof seven were previously undescribed [28]. Generally, the overlapping phenotypes for NAA10 patients are ID, DD and cardiac abnormalities. However, distinct phenotypes may also be correlated to specific effects of the different variants [25]. NAA10 polyadenylation signal variants [29], a splice-donor variant [30] and a small (4 bp) deletion in the penultimate exon [28] were found to cause Lenz microphthalmia syndrome (OMIM#309800) in males, while female carriers of the respective variants were unaffected in the described families.

Thus far, little is known about the exact disease mechanisms causative of disease in NAA10 patients. Here we describe a ten-year-old girl with a novel de novo NAA10 p.(His16Pro) missense variant and severe syndromic ID, severely delayed motor and language development and disturbed behavior with hyperactivity.

## Case presentation

### Patient description

**Fig. 1:**
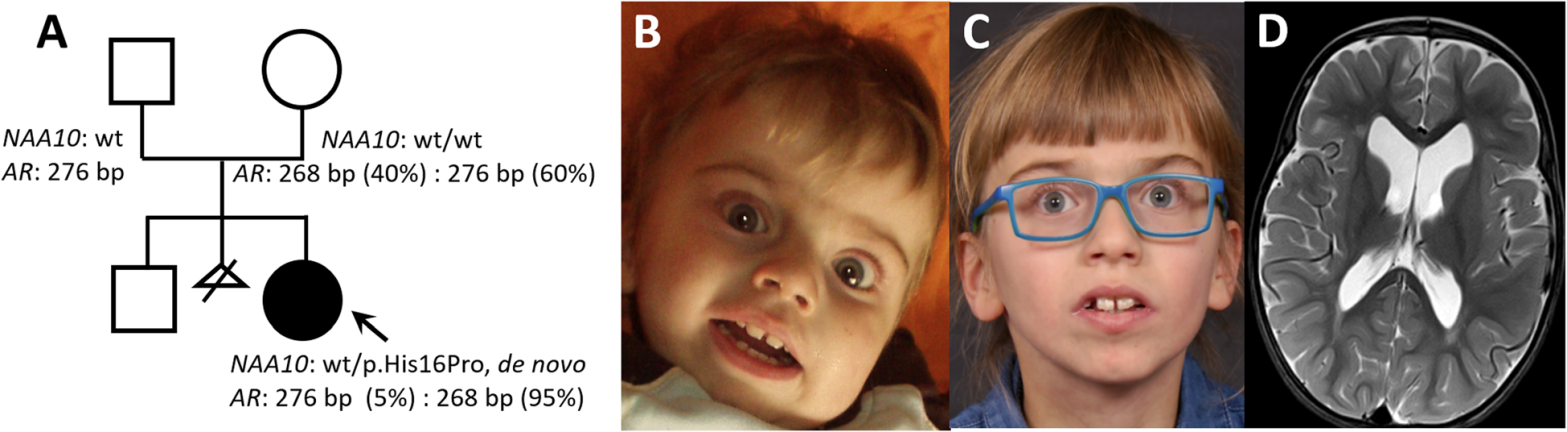
**a** Pedigree with *NAA10* genotypes, repeat length at the *AR*-locus and X-inactivation pattern. The *NAA10* mutation is absent in blood of the parents; the maternal X-inactivation pattern is random, the patient’s pattern is skewed with the paternal allele (276 p) being almost completely digested and the maternal allele (268 bp) almost undigested; **b** patient at the age of 1 year and 4 months and **c** 9 years and 6 months; **d** cMRI at the age of 2 years and 2.5 months shows a moderate dilatation of the ventricular system and extracerebral cerebrospinal fluid (CSF) spaces

The patient is a girl, now 10 years old, second child of a non-consanguineous couple of Austrian descent (Fig. 1a, b, c). The patient has a healthy older brother; one pregnancy was lost at an early stage. Parental age at delivery was 35 years each. The girl was born at term (39th week of gestation) by vaginal delivery - after manual turning from breech position in the 36th week of gestation. Birth weight was 3440 g (75th centile), length was 52 cm (75th centile) and birth occipital head circumference (OFC) was 34 cm (25th centile). She had club feet a small atrial septal defect (ASD) which resolved spontaneously later and a hip dysplasia (grade IIC – D). Postnatally, oxygen mask needed to be applied at night because of oxygen desaturations. Because of symptoms resembling interstitial lung disease (chronic tachydyspnea, recurrent pneumonia and bronchitis) a lung biopsy was performed at the age of 3 years without revealing any specific findings.

Breast feeding was initially possible without any problems. However, increasing feeding difficulties required feeding via PEG tube from the age of 7 months until the age of 3 years and 9 months.

At the age of 2 years and 2 months brain imaging (cMRI) showed moderate dilatation of ventricular system and extracerebral CSF spaces (Fig. 1d).

At the age of 3 years myopia (− 4.75 dpt) was noted.

At the age of 4 years she started to walk independently with a wide based gait; her height and OFC were in the lower normal range with 97 cm (25th centile) and 49 cm (2nd – 9th centile).

At her latest examination she was 9 years and 11 months of age and showed short stature with a height of 120 cm (0.4th centile, −3SD), and an OFC of 49 cm (< 0.4th centile, − 4 SD) indicating secondary microcephaly. She is very meagre with little subcutaneous fat. Her upper front teeth are broad and she has discrete facial dysmorphisms (thin horizontal eyebrows, slight hypertelorism, long palpebral fissures, wide mouth, deep nasal root, Fig. 1b, c).

At that age she had normal findings in cardiological investigations including normal ECG without arrhythmias and normal ultrasound investigation.

Skeletal findings include a progressive pectus excavatum, scoliosis, sandal gap and slightly broad end-phalanges of the thumb and first toe. The length of her legs is unequal, which is compensated for by heightened heels.

At the age of 10 years she is non verbal and is not able to understand words. A limited form of communication is possible using gestures. She is not diaper free yet. IQ was not formally tested, but ID can be described as severe.

Her behavior is extremely hyperactive with stereotypic actions, restlessness and a short attention span.

Whole exome sequencing (WES) revealed a novel heterozygous de novo missense-variant in *NAA10* (NM_003491.3) c.[47A > C];[=] (p.[His16Pro];[=]).

### NAA10 sequence- and structural analysis

**Fig. 2:**
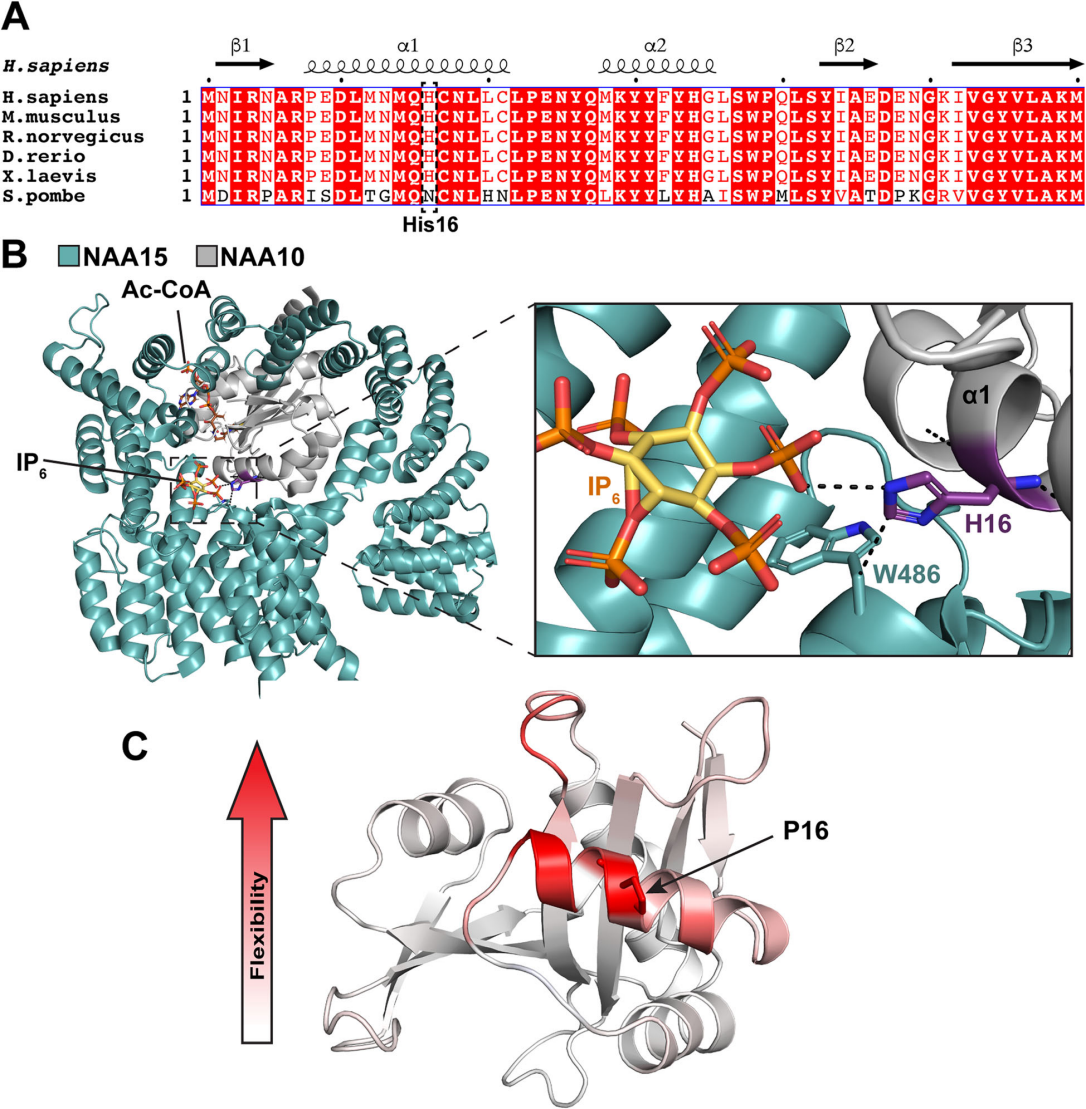
NAA10 multiple sequence alignment and structural analysis. **a** Multiple sequence alignment of NAA10 orthologues. Amino acid conservation is shown in red and secondary structure was based on the human NatA structure (PDB ID: 6C9M). **b** Human NatA structure (PDB ID: 6C9M) with Ac-CoA from *S. pombe* NAA10 structure (PDB ID: 4KVX). NAA15 is shown in teal, NAA10 is shown in grey, and IP_6_ and Ac-CoA are shown as sticks colored by atom. His16 (purple) is located in NAA10 α1 helix and was predicted by PyMOL to interact with IP_6_ and Trp486 of NAA15 (indicated by black dots). **c** The His16Pro mutation was predicted by DynaMut to increase structure flexibility (red) in the NAA10 α1 helix

A NAA10 multiple sequence alignment showed that His16 is conserved in *H. sapiens*, *M. musculus*, *R. norvegicus*, *D. rerio*, *X. laevis*, but not in *S. pombe*, suggesting that His16 may be important for protein function or stability in higher eukaryotes (Fig. 2a). A structural analysis of NatA revealed that His16 is situated in the α1 helix of NAA10 which is part of the NAA10-NAA15 binding interface (Fig. 2b). The side chain of His16 is facing outside of NAA10 towards NAA15 and the complex-bound ligand IP_6_. In silico interaction predictions in PyMOL revealed that His16 potentially interacts with IP_6_ and Trp486 of NAA15, indicating that His16 may be important for binding of NAA15 as well as IP_6_. Furthermore, the introduction of proline in position 16 was predicted by DynaMut to increase the flexibility in the α1 helix (Fig. 2c), most likely perturbing the interactions mediated by His16 and maybe also other interactions between NAA10 α1 helix and NAA15. Altogether, the in silico analyses indicate that the NAA10 p.(His16Pro) variant could hamper binding of NAA15 and the ligand IP_6_, potentially affecting NatA complex formation and catalytic activity.

### NatA complex formation and catalytic activity

**Fig. 3:**
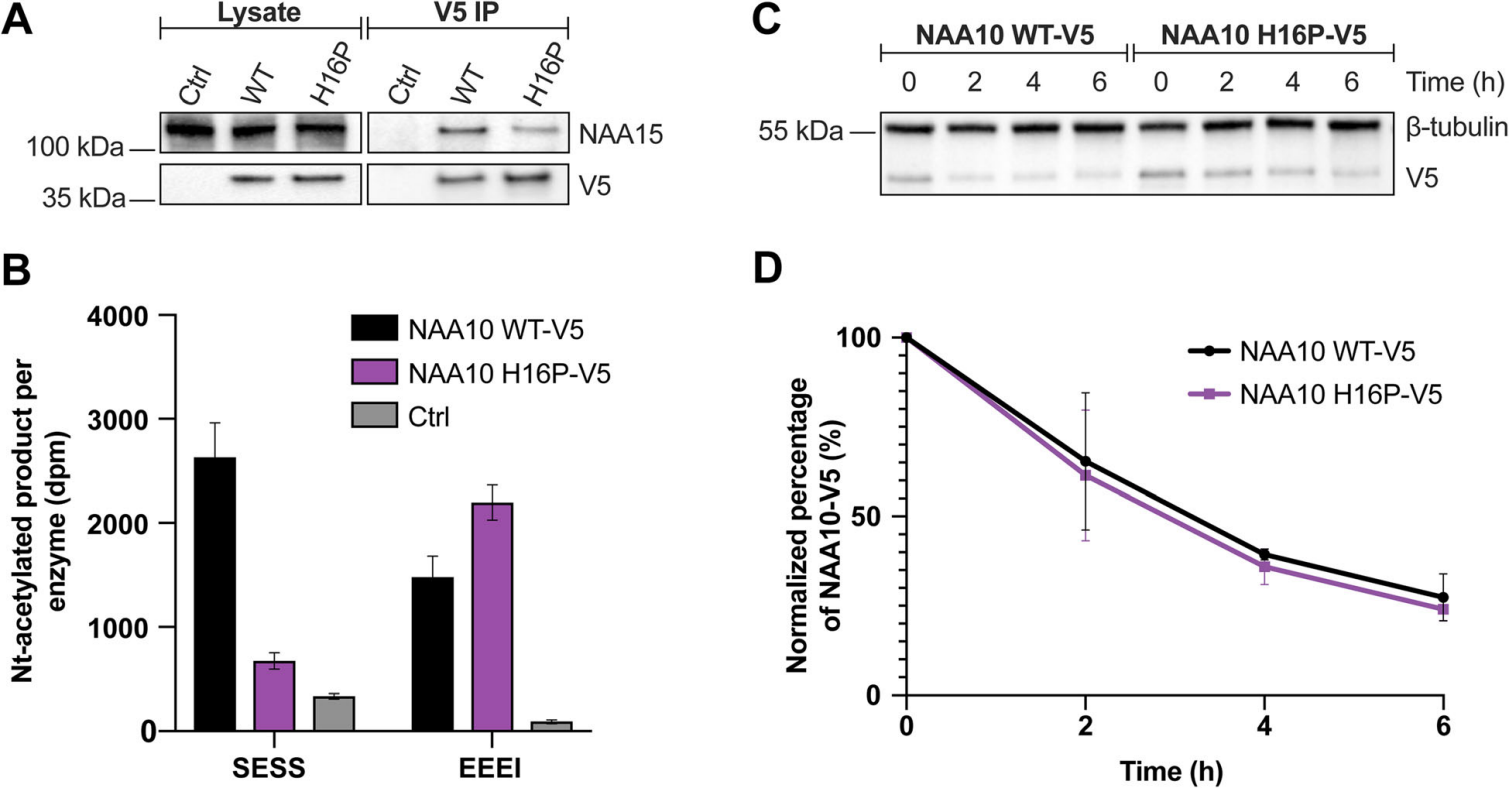
Impact of NAA10 H16P-V5 on NatA complex formation, enzymatic activity and cellular stability. **a** Western blot analysis of NAA10 WT-V5 and NAA10 H16P-V5 subjected to V5-immunoprecipitation from HeLa cells. V5- and NAA15 antibodies were used for protein detection and NAA10-V5 and NAA15 protein bands were quantified. **b** Nt-acetylation assay showing catalytic activity of NAA10 H16P-V5 and NAA10 WT-V5. The catalytic activity towards the NatA substrate SESS_24_ and monomeric NAA10 substrate EEEI_24_ was normalized to the amount of NAA15 and NAA10-V5, respectively. Negative controls contained either β-gal-V5 or no peptide. The Nt-acetylation assay shown is representative of three independent setups, each with three technical replicates. **c** Western blot analysis of cell lysates from 0 h (no treatment) and 2, 4 and 6 h after protein synthesis was inhibited by cycloheximide treatment. V5-tag antibody was used to detect NAA10-V5 protein and β-tubulin antibody was used for loading control. **d** Stability curve displaying the relative amount of NAA10-V5 present at each time point after cycloheximide treatment. The band intensities were quantified from the Western blot (C) and the band intensities of NAA10-V5 variants at each time point were normalized to both the loading control and time point 0 h of the respective variant. The stability curve is representative of three independent setups

To assess how the NAA10 p.(His16Pro) variant might affect NatA complex formation and the enzyme’s catalytic activity in comparison to NAA10 WT, V5-tagged NAA10 was immunoprecipitated from transfected HeLa cells. While NAA10-WT-V5 co-immunoprecipitated endogenous NAA15 as expected, NAA10 H16P-V5 in contrast pulled out less NAA15 (Fig. 3a). This suggests that the NAA10 p.(His16Pro) variant has impaired binding affinity for NAA15. Further, the immunoprecipitates were subjected to Nt-acetylation assays to measure catalytic activity (Fig. 3b). Nt-acetylation assays were performed using the NatA substrate SESS_24_ and the in vitro monomeric NAA10 substrate EEEI_24_. The measured catalytic activity towards SESS_24_ and EEEI_24_ was normalized to the amount of NAA15 (equals the amount of NatA complex) and NAA10-V5 (both monomeric and complexed state), respectively. As shown in Fig. 3b, NAA10 H16P-V5 displayed an approximately 4-fold decrease in NatA catalytic activity towards SESS_24_ as compared to NAA10 WT-V5. However, the monomeric NAA10 catalytic activity towards EEEI_24_ was increased 1.5-fold for NAA10 H16P-V5 relative to NAA10 WT-V5. Altogether the activity data indicate that the NAA10 p.(His16Pro) variant has aberrant NatA catalytic activity as well as impaired binding of NAA15, while the monomeric NAA10 catalytic activity appears to be intact.

### NAA10 WT-V5 and NAA10 H16P-V5 protein turnover

The cellular stabilities of NAA10 WT-V5 and NAA10 H16P-V5 were assessed by cycloheximide chase experiments. V5-tagged NAA10 variants were overexpressed in HeLa cells followed by cycloheximide treatment. The protein turnover was determined by Western blot analysis using antibodies against the V5-tag and β-tubulin as a loading control (Fig. 3c). As seen in Fig. 3d, the protein turnover rate was equal between NAA10 WT-V5 and NAA10 H16P-V5, suggesting that the NAA10 p.(His16Pro) variant does not affect the cellular stability of NAA10.

### Whole exome sequencing

Single whole exome sequencing and filtering of variants was performed as described previously [31]. The de novo status of the NAA10 variant was determined by targeted sanger sequencing of DNA from the parents. Paternity was confirmed by sanger sequencing of X-chromosomal SNPs.

### Multiple sequence alignment, structural analysis and databases

Clustal Omega [32] and ESPript 3.0 [33] was used to create a NAA10 multiple sequence alignment. PyMOL [34] and DynaMut [35] was used to visualize and study the human NatA structure (PDB ID: 6C9M) [36]. The human NatA structure was merged with acetyl-CoA from the *S. pombe* NAA10 structure (PDB ID: 4KVX) [6] in PyMOL. The NAA10 p.(His16Pro) variant was absent in 18,000 samples of the Munich exome server and absent in the ExAC and gnomAD databases.

### X-inactivation test and direction of skewing

X-inactivation status in DNA from leukocytes was determined according to standard protocols [37]. Length polymorphisms of the repeat in the AR gene were determined in DNA from blood of the father (276 bp), the mother (268 bp/276 bp) and the patient (268 bp/276 bp).

### Site directed mutagenesis

The NAA10 (NM_003491.3) c.47A > C p.(His16Pro) missense variant was introduced into a pcDNA3.1/*NAA10*-V5-His plasmid using Q5 Site Directed Mutagenesis Kit (New England Biolabs) with forward primer 5′-AACATGCAGCCCTGCAACCTC-3′ and reverse primer 5′-CATTAGGTCCTCTGGCCT-3′. The annealing temperature was 64 °C. The *NAA10* sequence was verified by sequencing.

### Transfection and immunoprecipitation

HeLa (ATCC® CCL-2™) were transfected 2 days prior to immunoprecipitation (IP) using X-tremeGENE 9 DNA Transfection Reagent (Roche). 10 × 10^6^ HeLa cells transfected with 6 μg of pcDNA3.1/*NAA10*-H16P-V5, 6 μg of pcDNA3.1/*LacZ*-V5 (control-IP) or 4 μg of pcDNA3.1/*NAA10*-WT-V5 and 2 μg of empty pcDNA3.1/V5 were used per IP sample. Harvested cells were lysed for 15 min at 4 °C in 1 ml IPH lysis buffer (50 mM Tris-HCl pH 8.0, 150 mM NaCl, 5 mM EDTA, 0.5% NP-40, 1× complete EDTA-free protease inhibitor cocktail (Roche)). Samples were then centrifuged (17000 *x g*, 4 °C) for 5 min to remove cell debris. The supernatants were incubated with 4 μg V5-tag antibody (Invitrogen, #R960–25) on a tube rotator for 2 h at 4 °C. Each sample was then mixed with 40 μl Dynabeads Protein G (Invitrogen). Following overnight incubation, the magnetic beads were washed three rounds in IPH lysis buffer and two rounds in acetylation buffer (50 mM Tris-HCl pH 8.5, 1 mM EDTA, 10% Glycerol). Finally, the IP samples were resuspended in 90 μl acetylation buffer and used in a Nt-acetylation assay. The IP samples were also analyzed by Western blot using V5-tag antibody (1:5000, Invitrogen, #R960–25) and NAA15 antibody (1:2000, BioGenes [3]). ChemiDoc XRS+ system (Bio-Rad) and Imagelab Software (Bio-Rad) were used for imaging and quantification of Western blots.

### In vitro Nt-acetylation assay

The catalytic activity of NAA10 WT-V5 and NAA10 H16P-V5 was tested in Nt-acetylation assays as described [38]. The 25 μl reaction mixtures consisted of 10 μl IP sample, 50 μM [^14^C]-Ac-CoA (Perkin-Elmer), 200 μM substrate peptide SESS_24_ (SESSSKSRWGRPVGRRRRPVRVYP) or EEEI_24_ (EEEIAALRWGRPVGRRRRPVRVYP) (BioGenes), and acetylation buffer.

Substrate peptide was substituted with acetylation buffer in negative controls. The reactions were incubated on a thermomixer (37 °C, 1400 rpm) and stopped after 30 min. Reaction mixtures were transferred to P81 phosphocellulose filter squares (Millipore). Filter squares were washed in 10 mM HEPES buffer (pH 7.4), dried and added to tubes with 5 ml Ultima Gold F scintillation cocktail (Perkin-Elmer). [^14^C]-acetyl signal was measured using a TriCarb 2900TR Liquid Scintillation Analyzer (Perkin-Elmer). The [^14^C]-acetyl signal for the reactions containing SESS_24_ and EEEI_24_ were normalized to the amount of NAA15 and NAA10-V5, respectively, after Western blot analysis.

### Cycloheximide chase experiment

NAA10 protein turnover was assessed by cycloheximide chase experiment as previously described [26]. In brief, 3 × 10^5^ HeLa cells (ATTC, CCL-2) per well were transfected with either 1.8 μg pcDNA3.1/*NAA10*-H16P-V5 or 1.2 μg pcDNA3.1/*NAA10*-WT-V5 and 0.6 μg empty pcDNA3.1/V5 using X-tremeGENE 9 DNA Transfection Reagent (Roche). Culture medium was replenished after 4 h and cells were grown for 2 days. To start the chase experiment, cells were subjected to cycloheximide (50 μg/ml) and then harvested at specific time points (0, 2, 4 and 6 h). Each time point sample was analyzed by Western blot using V5-tag antibody (1:5000, Invitrogen, #R960–25) and β-tubulin antibody (1:3000, Sigma, T5293). The amount of NAA10-V5 present at timepoints 2, 4 and 6 h post treatment was normalized to the amount of NAA10-V5 present at timepoint 0 h as well as β-tubulin as a loading control.

## Discussion and conclusions

Here we characterize a previously undescribed novel de novo NAA10 p.(His16Pro) missense variant in a ten-year-old female with severe syndromic intellectual disability, severely delayed motor and language development and hyperactive behavior.

In silico analyses were performed to investigate how the NAA10 p.(His16Pro) variant may affect intra- and intermolecular interactions (Fig. 2). His16 is strongly conserved in higher eukaryotes (Fig. 2a) and the α1 helix in which His16 is located mediates interactions important for binding of NAA15 [6]]. His16 was predicted to form interactions with both Trp486 of NAA15 and IP_6_ (Fig. 2b). IP_6_ is a ligand thought to have an evolutionarily conserved stabilizing role in NatA due to its identification in both human and *S. cerevisiae* NatA crystal structures [36, 39]. The His16Pro mutation introduces a proline with a cyclic side chain which has more conformational rigidity compared to other amino acids. Since the Cα-N bond of a proline in a peptide is incorporated in the ring-structure, the torsion angles ϕ and ψ of proline are not able to adopt a suitable α-helix formation. Furthermore, proline will typically introduce a destabilizing kink when located in the middle of α-helices due to its backbone’s inability to make hydrogen bonds. The introduction of proline in position 16 most likely destabilizes the α1 helix of NAA10 which may lead to increased flexibility in the α1 helix N- and C-terminus as well as close neighboring residues (Fig. 2c). Thus, the NAA10 p.(His16Pro) variant may debilitate binding of NAA15 and IP_6_ due to loss of interactions mediated by His16 as well as other NAA10 α1-helix residues due to increased local flexibility. Consequently, the NatA complex formation and/or catalytic activity of NAA10 p.(His16Pro) may be impaired.

The functional impact of NAA10 p.(His16Pro) was investigated in a Nt-acetylation assay using immunoprecipitated V5-tagged NAA10 H16P and NAA10 WT (Fig. 3a and b). In agreement with the in silico analyses, less NAA15 was co-immunoprecipitated with the NAA10 H16P-V5 variant as compared to NAA10 WT-V5, indicating that NAA10 p.(His16Pro) has a reduced binding affinity for NAA15. Furthermore, the Nt-acetylation assay revealed a 4-fold reduced catalytic activity of NAA10 H16P-V5 relative to NAA10 WT-V5 towards the NatA substrate SESS_24_ when normalized to the amount of NAA15 present in the reaction. Contrarily, the catalytic activity of NAA10 H16P-V5 towards the in vitro monomeric NAA10 substrate EEEI_24_ was increased as compared to NAA10 WT-V5. However, since NAA10 H16P-V5 pulled down less NAA15 compared to NAA10 WT-V5, the NAA10 H16P-V5 sample contained more monomeric NAA10. Monomeric NAA10 has a 1000-fold higher activity towards EEEI_24_ than NatA [9], and thus the actual monomeric NAA10 catalytic activity of NAA10 H16P-V5 and NAA10 WT-V5 is probably close to equal. In sum, these results suggest that the NatA complex formation and NatA catalytic activity of NAA10 p.(His16Pro) is impaired, whereas the monomeric NAA10 NAT activity is intact. The cycloheximide chase experiments revealed that NAA10 H16P-V5 did not have an altered cellular protein turnover compared to NAA10 WT-V5 (Fig. 3c and d) suggesting that the in vivo stability of this protein variant is not compromised.

*NAA10* is an X-linked gene, and males hemizygous for pathogenic NAA10 variants have generally been more severely affected compared to heterozygous females. In fact, several previously reported heterozygous females have shown mild to no symptoms due to skewed X-inactivation towards the disease allele [20–22, 24, 30]. The blood leukocyte X-inactivation pattern of the female reported herein was skewed (95/5) towards the maternally inherited X-chromosome. The determination of the parental allele that harbours the *NAA10*-variant was hampered by the lack of nearby SNPs in this family. Thus at the moment we do not know which is the parental allele the *NAA10*-variant in our patient is located on. Further experiments are necessary, e.g. using length polymorphisms of nearby microsatellites to address this question.

Despite the increasing number of pathogenic NAA10 variants reported, the underlying disease mechanisms remain elusive. Originally, the phenotype severity was thought to be directly linked to the reduction in catalytic activity [24]. However, this has been shown to be far more complex, and different NAA10 variants are likely to operate through a variation of disease mechanisms affecting different NAA10 functions [22, 25]. In conclusion, our in silico and functional characterization of the NAA10 p.(His16Pro) variant indicate that loss of NatA-mediated Nt-acetylation is causative of disease in the female reported herein. Further studies would be needed to determine whether KAT- or non-catalytic roles of NAA10 are also affected and contributing factors to disease.

## Data Availability

All data generated or analyzed during this study are included in this published article and its supplementary information files. Generated plasmids are available from the corresponding author on request. Information on the NAA10 missense variant c.[47A > C];[=] (p.[His16Pro];[=]) has been submitted to to the NCBI-Database ClinVar (ClinVar accession SCV001164597). Sequence analyses were performed using NCBI Reference Sequence database, GenBank: NM_003491.3.

## Abbreviations

NAA10: N-alpha acetyltransferase 10
NAA15: N-alpha acetyltransferase 15
NAT: N-terminal acetyltransferase
NatA: N-terminal acetyltransferase A
Ac-CoA: Acetyl coenzyme A
ID: Intellectual disability
DD: Developmental delay
WES: Whole exome sequencing
IP: Immunoprecipitation
